# Differences in ultrasound‐derived arterial wall stiffness parameters and noninvasive blood pressure between Friesian horses and Warmblood horses

**DOI:** 10.1111/jvim.15705

**Published:** 2020-02-07

**Authors:** Lisse Vera, Dominique De Clercq, Glenn Van Steenkiste, Annelies Decloedt, Koen Chiers, Gunther van Loon

**Affiliations:** ^1^ Department of Large Animal Internal Medicine, Faculty of Veterinary Medicine, Ghent University, Equine cardioteam Ghent University Merelbeke Belgium; ^2^ Department of Pathology, Bacteriology and Poultry Diseases, Faculty of Veterinary Medicine Ghent University Merelbeke Belgium

**Keywords:** compliance coefficient, distensibility coefficient, pulse wave velocity, stiffness index

## Abstract

**Background:**

Aortic rupture is more common in Friesians compared to Warmbloods, which might be related to differences in arterial wall composition and, as such, arterial wall stiffness (AWS). Currently, nothing is known about differences in AWS between these breeds.

**Objectives:**

Comparison of AWS parameters and noninvasive blood pressure between Friesians and Warmbloods.

**Animals:**

One hundred one healthy Friesians and 101 age‐matched healthy Warmbloods.

**Methods:**

Two‐dimensional and pulsed‐wave Doppler ultrasound examination was performed of the aorta, common carotid artery, and external iliac artery to define local and regional AWS parameters. Regional aortic AWS was estimated using aortic‐to‐external iliac artery pulse wave velocity (PWV_a‐e_) and carotid‐to‐external iliac artery pulse wave velocity (PWV_c‐e_). Noninvasive blood pressure and heart rate were recorded simultaneously.

**Results:**

Systolic, diastolic, and mean arterial blood pressure and pulse pressure were significantly higher in Friesians compared to Warmbloods. No significant difference in heart rate was found. Most local AWS parameters (diameter change, compliance coefficient, distensibility coefficient) were significantly lower in Friesians compared to Warmbloods, indicating a stiffer aorta in Friesians. This difference could be confirmed by the regional stiffness parameters. A higher PWV_a‐e_ and PWV_c‐e_ was found in Friesians. For the cranial and caudal common carotid artery and external iliac artery, most local AWS parameters were not significantly different.

**Conclusions and clinical importance:**

Results indicate that aortic AWS differs between Friesian and Warmblood horses. Friesians seem to have a stiffer aorta, which might be related to the higher incidence of aortic rupture in Friesians.

**List of Abbreviations**
ΔA: arterial lumen area change
ΔD: arterial diameter change
Ad: diastolic area
Ao: aorta
As: systolic area
AWS: arterial wall stiffness
CaCCA: caudal common carotid artery
CC_A_: compliance coefficient, calculated from lumen area measurements
CC_D_: compliance coefficient, calculated from diameter measurement
CO: cardiac output
CrCCA: cranial common carotid artery
CV: coefficient of variation
DAP: diastolic arterial pressure
DC_A_: distensibility coefficient, calculated from lumen area measurements
DC_D_: distensibility coefficient, calculated from diameter measurements
Dd: diastolic diameter
Ds: systolic diameter
EIA: external iliac artery
HR: heart rate
MAP: mean arterial pressure
PP: pulse pressure
PWV: pulse wave velocity
PWV_a‐e_: aortic‐to‐external iliac artery pulse wave velocity
PWV_c‐c_: caudal carotid‐to‐cranial carotid artery pulse wave velocity
PWV_c‐e_: carotid‐to‐external iliac artery pulse wave velocity
PWV_c‐f_: carotid‐to‐femoral artery pulse wave velocity
S_A_: arterial lumen area strain
SAP: systolic arterial pressure
S_D_: arterial diameter strain
SI: stiffness index
SV: stroke volume
SVR: systemic vascular resistance

## 1 INTRODUCTION

In Friesian horses, aortic rupture is more common compared to Warmblood horses.^1^ In contrast to Warmblood horses,^2^, ^3^, ^4^ aortic rupture in Friesian horses typically occurs close to the ligamentum arteriosum with formation of a pseudoaneurysm and aortopulmonary fistulation.^5^, ^6^ The reason why Friesians are predisposed to aortic rupture at this specific location remains unknown. An embryological defect occurring during fusion of the proximal aorta with the heart base has been hypothesized. However, valvular abnormalities, which would be expected with an embryological disorder, are not described in affected Friesians.^1^ In human medicine, aortic rupture usually is preceded by aortic aneurysm formation, which is not the case in Friesians.^7^ In human patients, elastin and especially collagen have been shown to be important factors contributing to aortic aneurysm formation.^8^, ^9^ Collagen fibrils, together with smooth muscle cells and elastin, form the primary load‐bearing components of the aortic wall. Both collagen and elastin are responsible for the tensile strength and the elasticity of the aortic wall. Collagen is essential to prevent over‐stretching and rupture, whereas elastin is essential for stretching and recoil of the artery, in order to buffer differences in pressure between systole and diastole.^9^ Previous postmortem studies identified differences in composition^10^, ^11^ and relative amounts^12^ of collagen and elastin between unaffected Friesian horses, affected Friesian horses and Warmblood horses, that might be related to the predisposition of Friesian horses to aortic rupture. Moreover medial necrosis is found at the site of rupture in affected Friesians,^7^ that might be related to connective tissue disorders,^13^ hypertension,^14^ ischemia,^6^ or inflammation.^15^ In addition to medial necrosis, disorganization and fragmentation of elastic laminae, aortic medial smooth muscle cell hypertrophy and accumulation of mucoid material also can be found histologically at the rupture site in affected Friesians.^7^

In human medicine, arterial wall stiffness (AWS) is known to be an independent predictor of cardiovascular mortality^16^, ^17^, ^18^, ^19^, ^20^ and can be assessed both regionally and locally. Measurement of carotid‐to‐femoral artery pulse wave velocity (PWV_c‐f_) generally is accepted as the most simple, noninvasive, robust, and reproducible method to determine regional AWS, and therefore is used as the gold standard method.^21^, ^22^, ^23^, ^24^, ^25^, ^26^ The pulse wave velocity (PWV) represents how fast a pressure wave travels over a certain length of the arterial tree. The pressure wave can be captured noninvasively using tonometry or pulsed wave Doppler ultrasound.^26^, ^27^ The PWV is calculated as the difference in traveled distance (ΔDist) between 2 recording sites (eg, carotid and femoral artery) divided by the time delay (Δt) between the 2 waveforms: PWV (m/s) = ΔDist (m)/Δt (s). The stiffer the artery, the higher the PWV. Ultrasonographic evaluation of lumen area or diameter change in relation to distending pressures can be used to estimate local AWS.^21^, ^24^, ^25^ Larger diameter and lumen area change indicate a more compliant artery.

To further understand the underlying cause of the predisposition of the Friesian breed to aortic rupture, we assessed both regional and local AWS parameters of the aorta, cranial, and caudal common carotid artery and external iliac artery in vivo, comparing Friesian horses to Warmblood horses. All AWS parameters were derived using vascular ultrasound examination. We hypothesized that Friesians would have stiffer arteries, which may predispose them to aortic rupture.

## 2 MATERIALS AND METHODS

Cardiovascular ultrasound examination was performed in 101 healthy Friesian horses (mean age ± SD, 12 ± 6 years) and 101 age‐matched, healthy Warmblood horses (mean age, 11 ± 5 years). Friesians consisted of 73 mares, 23 geldings, and 5 stallions. Warmblood horses consisted of 57 mares and 44 geldings. All horses were privately owned and informed consent was obtained. The study was approved by the ethical committee of the Faculty of Veterinary Medicine and Bioscience Engineering (EC2018/111). In every horse, height at the withers, tail circumference, chest circumference and length, defined as the length between the most cranial point of the shoulder and the tuber ischiadicum, was measured. Body weight was calculated as length × (chest circumference)^2^/11 900.^28^ In all horses, auscultation, echocardiography, and ECG were performed. Inclusion criteria were: no more than a 2/6 left‐ or right‐sided systolic or diastolic murmur on auscultation, no more than mild valvular regurgitation visible with Doppler ultrasound examination, and absence of atrial and ventricular premature beats during a 15‐minute ECG recording at rest.

### 2.1 Blood pressure

Systolic (SAP), diastolic (DAP), and mean (MAP) arterial blood pressure were measured noninvasively, simultaneously with acquisition of ultrasound images, using an oscillometric device (Cardell Veterinary Monitor, 9401, Midmark), with a cuff placed around the base of the tail. To correct readings to the level of the heart base, a correction factor of 0.77 mmHg was added for every cm in difference between the right atrium (estimated at the height of the shoulder) and the cuff.^29^ As recommended,^30^ the mean of 5 consecutive, consistent measurements was taken. In all horses, a cuff width of 9 cm was used. Tail circumference was measured to calculate the cuff width‐to‐tail circumference ratio. A cuff width‐to‐tail circumference ratio between 0.4 and 0.6^31^, ^32^ was achieved in all horses except for 3 Friesians in which tail circumference was slightly too large, resulting in a cuff width‐to‐tail circumference ratio of 0.3.

### 2.2 Ultrasound

Ultrasound imaging was performed (Vivid IQ, GE Healthcare) in the standing, nonsedated horse. Images only were collected with a heart rate (HR) <50 beats per minute. Stroke volume (SV) was calculated as pulsed wave Doppler‐derived aortic velocity time integral (left parasternal view) multiplied by aortic valve cross‐sectional area (right parasternal view).^33^ Cardiac output (CO) was determined as SV × HR.

### 2.3 Ultrasonographic measurements

#### 2.3.1 B‐ and M‐mode images

**Figure 1:**
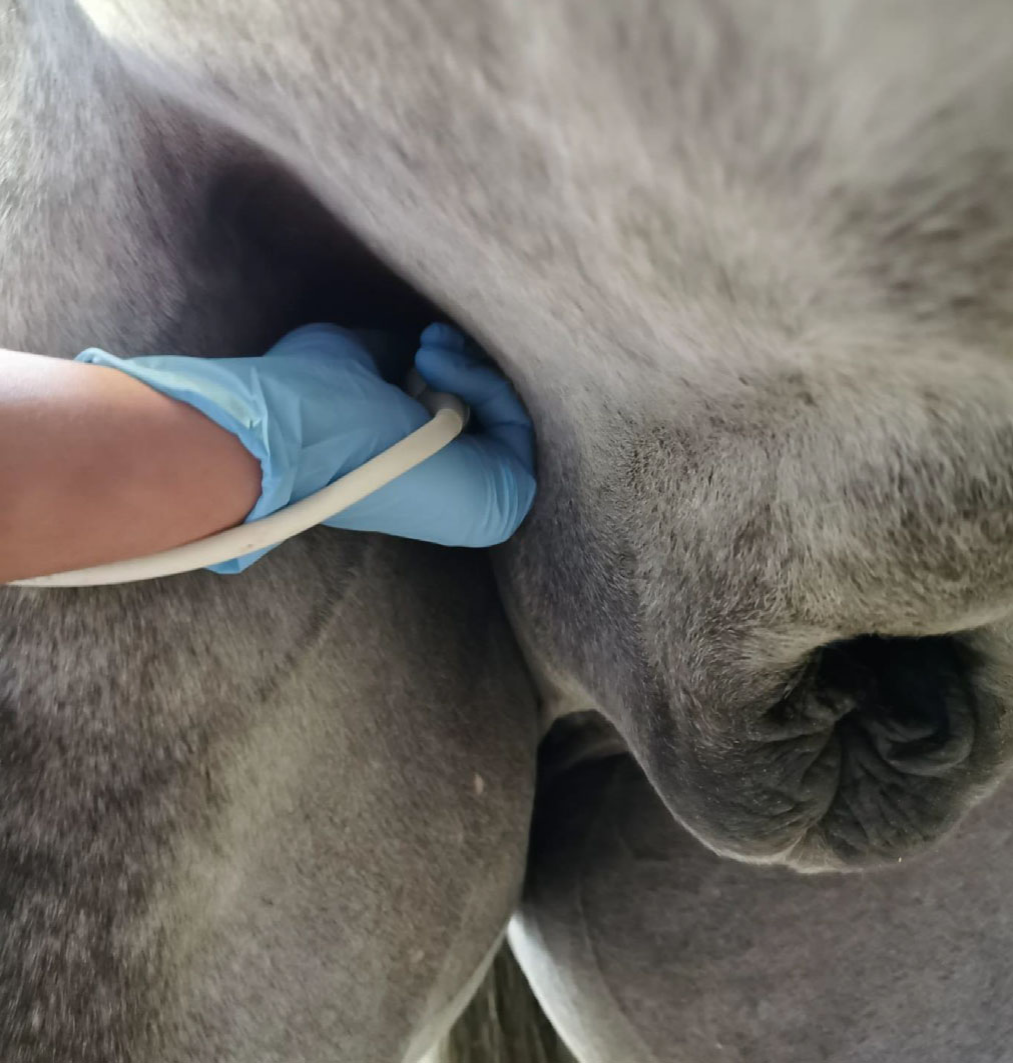
Exact probe location to collect images of the external iliac artery from the inguinal region

Ultrasound examination was performed from 4 different arterial locations to calculate AWS: the aorta from a right parasternal position, the right caudal common carotid artery (15 cm cranial to the thoracic inlet), the right cranial common carotid artery (30 cm more cranially) and the right external iliac artery, from the inguinal region (Figure 1) just proximal to the side branch, deep femoral artery (arteria profunda femoris). For the aorta, a 1.5 to 4.6 MHz phased array probe (M5Sc‐RS, GE Healthcare) was used to obtain a right parasternal left ventricular outflow tract view (frequency 4.0 MHz/8 MHz, depth 26 cm, gain 10 dB). A 3.5 to 10 MHz linear transducer (9 L‐RS, GE Healthcare) was used to obtain cross‐sectional B‐ and M‐mode images from the cranial and caudal carotid artery (frequency 4.0 MHz/8.0 MHz, depth 4 cm, gain 16 dB). For the external iliac artery, a 2.7 to 8 MHz phased array probe (6S‐RS,GE Healthcare) was used to obtain longitudinal M‐mode images and cross‐sectional B‐mode images (gain 3.5 MHz/7.0 MHz, depth 7 cm, gain 4 dB). All images were stored for off‐line analysis (Echopac version 203, GE Healthcare).

#### 2.3.2 Pulsed wave Doppler

The pulse wave was captured noninvasively using pulsed wave Doppler. For the carotid and external iliac artery, a 2.7 to 8 MHz phased array probe (6S‐RS,GE Healthcare, frequency: 4 MHz, depth 7 cm, gain 5 dB, sample size 5 mm) was used and a 1.5 to 4.6 MHz phased array probe (M5Sc‐RS GE Healthcare, frequency 2 MHz, depth 26 cm, gain 10 dB, sample size 5 mm) was used for the aorta (left parasternal view). At all locations, angle correction was set at 45° and images were optimized to align with flow direction. All images were stored for off‐line analysis (Echopac version 203, GE Healthcare).

### 2.4 Local measurements of arterial wall stiffness

**Figure 2:**
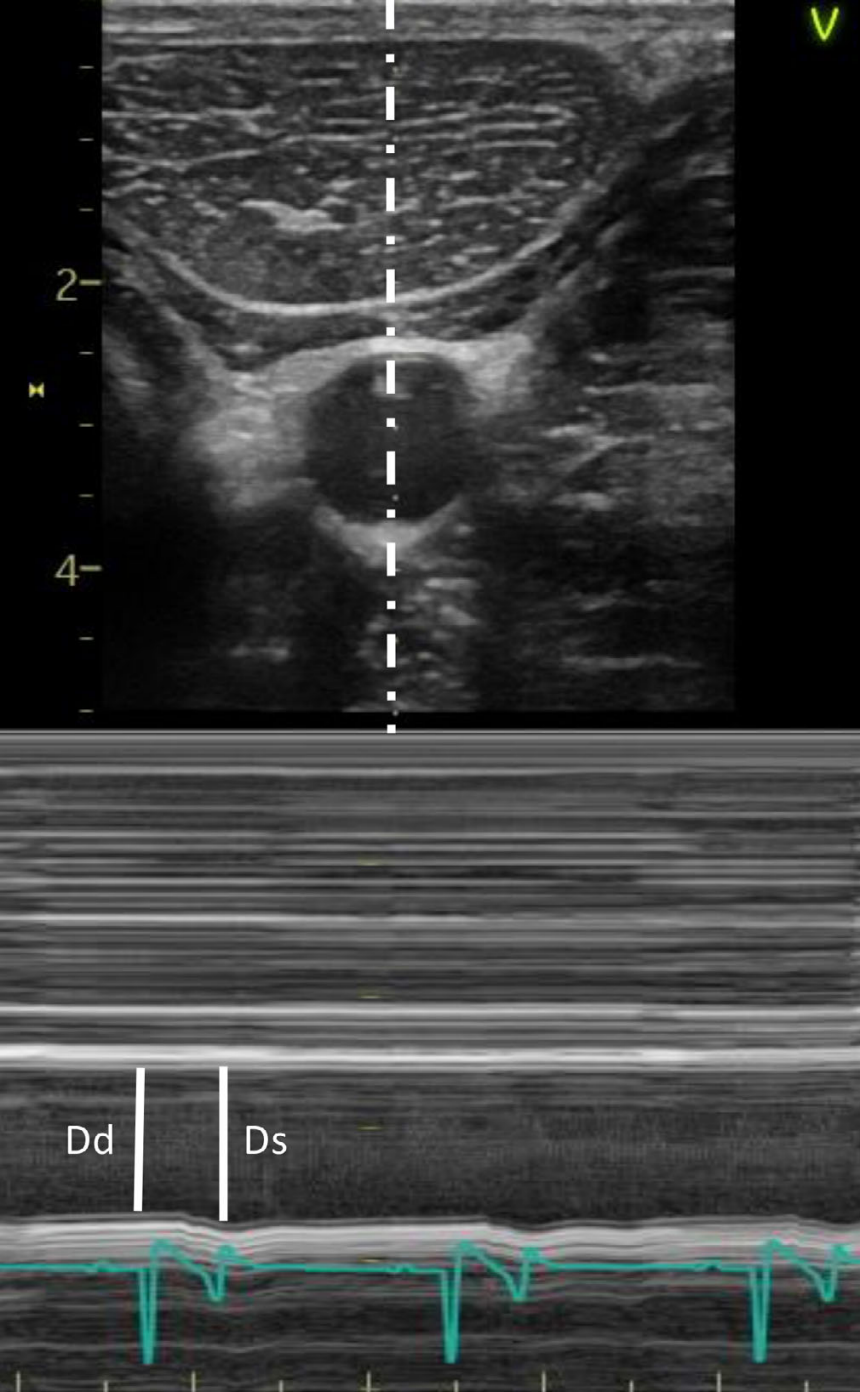
M‐mode systolic (Ds) and diastolic (Dd) diameter measurement at the level of the cranial common carotid artery. Diastolic measurements were performed at the time of the R peak of the simultaneously recorded ECG and systolic measurements were performed at maximal dilatation

Aortic diameters were measured at the sinotubular junction from a right parasternal left ventricular outflow tract view. Mean aortic diameters were calculated from 3 consecutive cycles. Diameters were measured from cross‐sectional M‐mode images for the cranial and caudal common carotid artery. On the other hand, areas were measured from cross‐sectional B‐mode images. For the external iliac artery, diameters were measured from longitudinal M‐mode images and areas from cross‐sectional B‐mode images. Mean carotid and external iliac artery diameters were calculated from 3 times 3 consecutive heart cycles (9 in total). For all arteries, diastolic measurements were performed on the R peak of the simultaneously recorded ECG (base‐apex configuration) and systolic measurements were performed at maximal dilatation (Figure 2).

#### 2.4.1 Arterial diameter and lumen area change

The change in diameter or lumen area between systole (s) and diastole (d) is defined as the arterial diameter change (ΔD = Ds − Dd) or lumen area change (ΔA = As − Ad), respectively.

#### 2.4.2 Arterial diameter and lumen area strain

The relative change in diameter or lumen area during the cardiac cycle is defined as the arterial diameter strain (S_D_ = ΔD/Dd) or lumen area strain (S_A_ = ΔA/Ad)_,_ respectively.

#### 2.4.3 Arterial distensibility

The relative diameter or area change for a pressure increment is defined as the arterial distensibility coefficient (DC_D_ = [ΔD/Dd]/PP; DC_A_ = [ΔA/Ad]/PP, respectively). Pulse pressure (PP) is defined as SAP − DAP.

#### 2.4.4 Arterial compliance

The absolute diameter/area change for a pressure increment is defined as the arterial compliance coefficient (CC_D_ = ΔD/PP; CC_A_ = ΔA/PP, respectively).

#### 2.4.5 Stiffness index

The ratio of the natural logarithm (systolic/diastolic pressure) to the relative change in diameter is defined as the stiffness index (SI = ln [SAP/DAP]/[ΔD/Dd]).

### 2.5 Regional measurements of arterial wall stiffness

**Figure 3:**
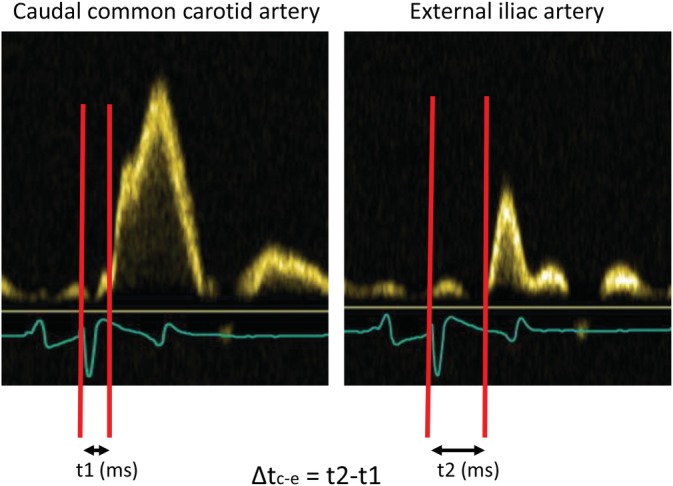
Measurement of the time it takes for the pressure wave to travel from the caudal carotid artery to the external iliac artery using pulsed wave Doppler. The time delay between the R wave of the synchronized ECG and the onset of the waveform is measured both at the level of the caudal common carotid artery (t1) and at the level of the external iliac artery (t2). To calculate the difference in time delay (Δt), the time delay at the location nearest to the heart (shortest time delay, t1) was subtracted from the mean time delay at the location the furthest away from the heart (longest time delay, t2)

The time delay (ms) between the R wave of the synchronized ECG and the onset (foot) of the waveform, captured using pulsed wave Doppler, was measured at every investigated location. Means were calculated from 3 times 3 consecutive beats (9 in total). The mean time delay at the location nearest to the heart (shortest time delay) was subtracted from the mean time delay at the location the farthest away from the heart (longest time delay) to calculate the difference in time delay (Δt; Figure 3).

#### 2.5.1 Caudal carotid‐to‐cranial carotid artery pulse wave velocity

The velocity at which the pressure wave travels between the caudal and cranial common carotid artery is defined as the caudal carotid‐to‐cranial carotid artery pulse wave velocity (PWV_c‐c_ [m/s] = ΔDist_c‐c_ [m]/Δt _c‐c_ [s]). A fixed distance of 0.3 m between both measuring places (ΔDist_c‐c_) was taken.

#### 2.5.2 Aorta‐to‐external iliac artery pulse wave velocity

The velocity at which the pressure wave travels between the aorta and the external iliac artery is defined as the aorta‐to‐external iliac artery pulse wave velocity (PWV_a‐e_ [m/s] = ΔDist_a‐e_ [m]/Δt_a‐e_ [s]). The ΔDist_a‐e_ represents the distance between the aortic valve and the external iliac artery. This distance was estimated based on measurements performed on 5 Warmblood horses (mean ± SD body weight, 633 ± 112 kg; height at the withers, 166 ± 10 cm) at necropsy. The distance between the aortic valve and the external iliac artery was measured and related to the length of the horse (measured from the most cranial point of the shoulder to the tuber ischiadicum). The mean value for this distance estimate was 0.62 times the length of the horse (range, 0.55‐0.66). Therefore, ΔDist_a‐e_ (m) = length of the horse (m) × 0.62.

#### 2.5.3 Carotid‐to‐external iliac artery pulse wave velocity

The velocity at which the pressure wave travels between the caudal common carotid artery and the external iliac artery is defined as the carotid‐to‐external iliac artery pulse wave velocity (PWV_c‐e_ [m/s] = ΔDist_c‐e_ [m]/Δt_c‐e_ [s]). The ΔDist_c‐e_ represents the difference in distance relative to the aortic root between the caudal carotid and external iliac arteries. To estimate this distance, in the same 5 horses at necropsy, the distance between the aortic valve and the caudal carotid artery was measured and was found to average 0.40 m (range, 0.37‐0.52 m). Because the pressure wave simultaneously travels from the aortic valve cranially (toward the caudal carotid artery) and caudally (toward the external iliac artery), this distance was subtracted from the estimated distance between the aorta and external iliac artery. Therefore, ΔDist_c‐e_ (m) = length of the horse (m) × 0.62‐0.40 m.

### 2.6 Statistical analysis of data

Normality of all variables and derived AWS parameters was evaluated graphically. Arterial diameters and areas, arterial blood pressures, PWV, HR, and CO were compared between Friesians and Warmbloods using an independent samples *t* test. For derived local stiffness values, a univariate model was built including breed (Friesian or Warmblood) and location (cranial carotid artery, caudal carotid artery, aorta and external iliac artery) as fixed factors and horse as random factor. Interaction was found between location and breed, therefore a univariate model, including location*breed as a fixed factor and horse as a random factor with post hoc Bonferroni correction for multiple comparisons was applied. *P* values <.05 were considered significant.

## 3 RESULTS

Although all horses tolerated the procedure well, images of the external iliac artery, especially transverse images, were the most difficult to obtain. The distance of 0.3 m between the caudal and cranial common carotid artery was difficult to define, because of the oblique probe position needed to align with flow direction.

Friesians were significantly less tall (height at the withers [mean ± SD], 162 ± 4 cm versus 167 ± 5 cm; *P* < .001), but longer (length of the horse, 188 ± 11 cm versus 180 ± 9 cm; *P* < .001), with a larger chest circumference (199 ± 7 cm versus 195 ± 8 cm; *P* < .001) and a larger calculated weight (627 ± 61 kg versus 575 ± 63 kg; *P* < .001) compared to Warmblood horses.

Friesians had significantly (*P* < .001) higher SAP (146 ± 18 mmHg versus 135 ± 14 mmHg), DAP (97 ± 12 versus 91 ± 10 mmHg), MAP (115 ± 15 mmHg versus 106 ± 13 mmHg), and PP (49 ± 9 mmHg versus 44 ± 9 mmHg) in comparison with Warmblood horses. No significant differences in HR or CO were found. Results are presented in Table 1. The mean tail circumference of the Friesians horses was significantly higher (25 ± 1 cm) in comparison with the Warmblood horses (22 ± 2 cm; *P* < .001), but both were within the reference range for the cuff used (17‐25 cm). The only exceptions were 3 Friesians for which tail circumference was slightly too large for the cuff used (26 cm in 1 horse and 27 cm in 2 horses).

**Table 1:** Comparison of systolic, diastolic, and mean arterial blood pressure and pulse pressure in Friesian horses and Warmblood horses

	Friesian (mean ± SD)	Warmblood (mean ± SD)	*P*‐value
SAP (mmHg)	**146 ± 18**	**135 ± 14**	**<.001**
DAP (mmHg)	**97 ± 12**	**91 ± 10**	**<.001**
MAP (mmHg)	**115 ± 15**	**106 ± 13**	**<.001**
PP (mmHg)	**49 ± 9**	**44 ± 9**	**<.001**
HR (bpm)	37 ± 5	36 ± 6	.14
CO (L/min)	34.18 ± 7.66	36.32 ± 8.88	.09

Abbreviations: CO, cardiac output; DAP, diastolic arterial pressure; HR, heart rate; MAP, mean arterial pressure; PP, pulse pressure; SAP, systolic arterial pressure.

Bold values indicate significant difference between Friesians and Warmbloods.

All measured arterial diameters and areas are presented in Table 2. Overall, arterial diameters and areas of all investigated arteries were smaller (2%‐20%) in Friesian horses compared to Warmblood horses. A detailed overview of all derived AWS parameters is found in Table 3. The AWS parameters indicate that Friesian horses might have a stiffer aorta. The PWV_a‐e_ and PWV_c‐e_, both regional aortic AWS parameters covering a large part of the aorta, were significantly higher in Friesian horses compared to Warmblood horses, indicating a stiffer aorta. The arterial diameter change, distensibility coefficient and the compliance coefficient, all 3 local aortic AWS parameters, were significantly lower in Friesian horses compared to Warmblood horses, also suggesting a stiffer aorta. For the cranial and caudal common carotid artery and the external iliac artery, most local AWS parameters were not significantly different between breeds, except for a higher diameter strain of the cranial carotid artery, a higher area strain of the caudal common carotid artery and a lower stiffness index of the external iliac artery in Friesians compared to Warmblood horses.

**Table 2:** Comparison of internal diastolic and systolic diameters and areas of the aorta, the cranial and caudal common carotid artery and the external iliac artery between Friesian horses and Warmblood horses

	Friesian (mean ± SD)	Warmblood (mean ± SD)	*P*‐value
Dd(Ao) (mm)	**59 ± 5**	**62 ± 5**	**.001**
Ds(Ao) (mm)	**65 ± 6**	**68 ± 6**	**<.001**
Dd(CrCCA) (mm)	**10.1 ± .9**	**10.5 ± .9**	**.006**
Ds(CrCCA) (mm)	10.8 ± 1.0	11.00 ± 1.0	.13
Ad(CrCCA) (mm^2^)	**78.1 ± 14.6**	**84.8 ± 18.6**	**.005**
As(CrCCA) (mm^2^)	**86.8 ± 16.0**	**93.1 ± 20.2**	**.02**
Dd(CaCCA) (mm)	**11.6 ± 1.1**	**11.9 ± 1.4**	**.04**
Ds(CaCCA) (mm)	12.5 ± 1.1	12.8 ± 1.4	.10
Ad(CaCCA) (mm^2^)	**108.8 ± 19.62**	**120.1 ± 26.0**	**.001**
As(CaCCA) (mm^2^)	**122.8 ± 20.9**	**132.3 ± 27.6**	**.01**
Dd(EIA) (mm)	**10.8 ± 1.1**	**11.8 ± 1.2**	**<.001**
Ds(EIA) (mm)	**11.2 ± 1.1**	**12.2 ± 1.2**	**<.001**
Ad(EIA) (mm^2^)	**107.2 ± 21.3**	**133.3 ± 28.9**	**<.001**
As(EIA) (mm^2^)	**116.9 ± 22.4**	**143.4 ± 30.5**	**<.001**

Abbreviations: Ad, diastolic area; Ao, aorta; As, systolic area; CaCCA, caudal common carotid artery; CrCCA, cranial common carotid artery; Dd, diastolic diameter; Ds, systolic diameter; EIA, external iliac artery.

Bold values indicate significant difference between Friesians and Warmbloods.

**Table 3:** Comparison of arterial wall stiffness parameters for the aorta, the cranial and caudal common carotid artery and the external iliac artery between 101 Friesian horses and 101 Warmblood horses

	Friesian (mean ± SD)	Warmblood (mean ± SD)	*P*‐value
Regional stiffness parameters			
PWV_a‐e_ (m/s)	**6.52 ± .90**	**5.95 ± .94**	**<.001**
PWV_c‐e_ (m/s)	**7.06 ± 1.60**	**5.79 ± 1.43**	**<.001**
PWV_c‐c_ (m/s)	7.96 ± 3.16	8.02 ± 3.58	.92
Local stiffness parameters			
Aorta			
ΔD(Ao) (mm)	**5.8 ± 2.1**	**6.3 ± 2.0**	.**01**
S_D_(Ao)	9.9 × 10^−02^ ± 3.9 × 10^−02^	10.3 × 10^−02^ ± 3.5 × 10^−02^	1.000
DC_D_(Ao) (/mmHg)	**2.1 × 10** ^**−03**^ ** ± 1.0 × 10** ^**−03**^	**2.5 × 10** ^**−03**^ ** ± 1.0 × 10** ^**−03**^	**.01**
CC_D_(Ao) (mm/mmHg)	**1.2 × 10** ^**−01**^ ** ± .6 × 10** ^**−01**^	**1.5 × 10** ^**−01**^ ** ± .6 × 10** ^**−01**^	**<.001**
SI (Ao)	4.9 ± 2.5	4.4 ± 2.2	1.000
Cranial carotid artery			
ΔD(CrCCA) (mm)	6.8 × 10^−01^ ± 2.7 × 10^−01^	5.3 × 10^−01^ ± 2.2 × 10^−01^	1.000
S_D_(CrCCA)	**6.8 × 10** ^**−02**^ ** ± 2.8 × 10** ^**−02**^	**5.1 × 10** ^**−02**^ ** ± 2.1 × 10** ^**−02**^	**<.001**
DC_D_(CrCCA) (/mmHg)	1.5 × 10^−03^ ± .7 × 10^−03^	1.2 × 10^−03^ ± .7 × 10^−03^	.14
CC_D_(CrCCA) (mm/mmHg)	1.5 × 10^−02^ ± .7 × 10^−02^	1.3 × 10^−02^ ± .7 × 10^−02^	1.000
SI (CrCCA)	7.1 ± 3.9	9.6 ± 5.9	.44
ΔA(CrCCA) (mm^2^)	8.7 ± 3.5	8.4 ± 3.8	1.000
S_A_(CrCCA)	1.1 × 10^−01^ ± .4 × 10^−01^	1.0 × 10^−01^ ± .4 × 10^−01^	1.000
DC_A_(CrCCA) (/mmHg)	2.5 × 10^−03^ ± 1.1 × 10^−03^	2.4 × 10^−03^ ± 1.3 × 10^−03^	1.000
CC_A_(CrCCA) (mm^2^/mmHg)	1.9 × 10^−01^ ± 1.0 × 10^−01^	2.1 × 10^−01^ ± 1.2 × 10^−01^	1.000
Caudal carotid artery			
ΔD(CaCCA) (mm)	9.3 × 10^−01^ ± 2.9 × 10^−01^	8.7 × 10^−01^ ± 3.3 × 10^−01^	1.000
S_D_(CaCCA)	8.2 × 10^−02^ ± 2.7 × 10^−02^	7.4 × 10^−02^ ± 3.0 × 10^−02^	.89
DC_D_(CaCCA) (/mmHg)	1.7 × 10^−03^ ± .7 × 10^−03^	1.8 × 10^−03^ ± .9 × 10^−03^	1.000
CC_D_(CaCCA) (mm/mmHg)	2.0 × 10^−02^ ± .7 × 10^−02^	2.1 × 10^−02^ ± 1.0 × 10^−02^	1.000
SI(CaCCA)	5.6 ± 2.6	7.1 ± 9.2	1.000
ΔA(CaCCA) (mm^2^)	14.0 ± 5.5	12.2 ± 5.4	1.000
S_A_(CaCCA)	**1.3 × 10** ^**−01**^ ** ± .5 × 10** ^**−01**^	**1.0 × 10** ^**−01**^ ** ± .5 × 10** ^**−01**^	**<.01**
DC_A_(CaCCA) (/mmHg)	2.8 × 10^−03^ ± 1.4 × 10^−03^	2.5 × 10^−03^ ± 1.3 × 10^−03^	1.000
CC_A_(CaCCA) (mm^2^/mmHg)	3.0 × 10^−01^ ± 1.5 × 10^−01^	2.9 × 10^−01^ ± 1.5 × 10^−01^	1.000
E × ternal iliac artery			
ΔD(EIA) (mm)	3.9 × 10^−01^ ± 1.6 × 10^−01^	3.9 × 10^−01^ ± 1.8 × 10^−01^	1.000
S_D_(EIA)	3.6 × 10^−02^ ± 1.5 × 10^−02^	3.3 × 10^−02^ ± 1.6 × 10^−02^	1.000
DC_D_(EIA) (/mmHg)	7.8 × 10^−04^ ± 3.5 × 10^−04^	7.9 × 10^−04^ ± 4.5 × 10^−04^	1.000
CC_D_(EIA) (mm/mmHg)	8.2 × 10^−03^ ± 3.8 × 10^−03^	9.3 × 10^−03^ ± 5.0 × 10^−03^	1.000
SI(EIA)	**12.3 ± 10.6**	**16.5 ± 14.8**	**.02**
ΔA(EIA) (mm^2^)	9.7 ± 4.8	10.1 ± 3.8	1.000
S_A_(EIA)	9.4 × 10^−02^ ± 5.1 × 10^−02^	7.7 × 10^−02^ ± 2.9 × 10^−02^	.99
DC_A_(EIA) (/mmHg)	2.0 × 10^−03^ ± 1.3 × 10^−03^	1.9 × 10^−03^ ± .8 × 10^−03^	1.000
CC_A_(EIA) (mm^2^/mmHg)	2.0 × 10^−01^ ± 1.2 × 10^−01^	2.4 × 10^−01^ ± 1.3 × 10^−01^	1.000

Abbreviations: ΔA, arterial lumen area change; ΔD, arterial diameter change; Ao, Aorta; CaCCA, caudal common carotid artery; CC_A_, arterial compliance coefficient calculated from lumen area change; CC_D_, arterial compliance coefficient, calculated from diameter change; CrCCA, cranial common carotid artery; DC_A_, arterial distensibility coefficient calculated from area change; DC_D_, arterial distensibility coefficient calculated from diameter change; EIA, e×ternal iliac artery; PWV_a‐e_, aorta to e×ternal iliac artery pulse wave velocity; PWV_c‐c_, caudal carotid to cranial carotid artery pulse wave velocity; PWV_c‐e_, carotid to e×ternal iliac artery pulse wave velocity; S_A_, arterial lumen area strain; S_D_, arterial diameter strain; SI, stiffness inde×.Bold values indicate significant difference between Friesians and Warmbloods.

## 4 DISCUSSION

In our study, both regional and local AWS parameters were determined echocardiographically as described previously.^34^ Regional AWS parameters describe the stiffness of an artery over a certain length, whereas local AWS parameters consider only a specific location of the arterial tree. In our study, echocardiographic measurements were performed at 4 arterial locations that met the following criteria: large, superficial arteries that are relatively easy to image using ultrasound. Therefore, the proximal aorta, the cranial and caudal common carotid artery and the external iliac artery were chosen. Regional AWS parameters were only assessed for the aorta and the common carotid artery because a large distance between 2 measuring sites, preferably on the same artery, is necessary to measure PWV reliably.^25^

The aorta is of major interest when assessing AWS because the aorta makes a substantial contribution to the arterial buffering capacity.^35^ In this study, both regional and local AWS parameters indicated a stiffer aorta in Friesian horses compared to Warmbloods. In accordance with the PWV_c‐f_ in human medicine, in our study the PWV_c‐e_ was calculated to assess the stiffness of the aorta. Although in human medicine PWV_c‐f_ is used as the gold standard method, we also calculated the PWV_a‐e_ to more accurately assess aortic stiffness. The PWV_c‐e_ assumes that the pulse wave travels at the same speed cranially (toward the carotid artery) and caudally (toward the external iliac artery), whereas our study shows that stiffness, and thus the velocity of the pulse wave, can differ substantially among different arteries. The PWV_a‐e_ on the other hand considers only the aorta itself and a very small part of the external iliac artery. Ideally, the aortic PWV should be measured between the proximal aorta and the aortic bifurcation into the external and internal iliac arteries. To examine the aortic bifurcation, transrectal ultrasonography would need to be performed. However, to avoid effects of stress on HR and BP, no transrectal ultrasound examination was performed, and the external iliac artery was examined instead. The PWV_a‐e_ as well as PWV_c‐e_ were significantly higher in Friesian horses compared to Warmbloods, indicating a stiffer aorta in Friesians. These higher PWV were not caused by a higher HR,^36^ because the HR in Friesians was not significantly different from that of Warmbloods. The stiffer aorta could be confirmed by local AWS parameters of the aorta: arterial diameter change, arterial compliance coefficient, and arterial distensibility coefficient all were lower in Friesians. Arterial diameter strain also was lower and the stiffness index was higher in Friesians compared to Warmblood, but not significantly. For the local AWS parameters of the cranial and caudal common carotid artery and the external iliac artery, most parameters did not differ significantly, indicating no difference in AWS of these vessels between Friesians and Warmbloods. Also the PWV_c‐c_, describing the regional AWS of the common carotid artery, was not significantly different between the breeds. These results suggest that in Friesians a difference in AWS is only present at the aortic level.

Systolic arterial blood pressure, DAP, MAP, and PP were significantly higher in Friesian horses compared to Warmblood horses. Therefore, AWS parameters, especially those that do not include pressure, should be interpreted with caution. Whether the higher blood pressure in Friesians was the cause or the consequence of stiffer arteries cannot be determined from our results. In human medicine, primary hypertension is known to lead to an increase in collagen density, associated with an increase in AWS.^9^, ^37^ Although the results found in our study did not indicate true hypertension, the continuously increased blood pressure in Friesians might lead to an increased amount of collagen in the Friesian aortic wall. Indeed, Friesians are known to have an increased amount of collagen in the tunica media of the aorta compared to Warmblood horses,^12^ in combination with a different collagen cross‐linking pattern.^10^, ^11^ As previously shown, an oversized cuff will underestimate blood pressure, whereas using too small a cuff in relation to tail circumference will overestimate it.^38^, ^39^ In our study, tail circumference was significantly larger in Friesian horses (24.5 ± 1.1 cm) compared to Warmblood horses (22.3 ± 1.7 cm). The cuff bladder used was suitable for a tail circumference of 17 to 25 cm, ensuring a width‐to‐tail circumference ratio between 0.4 and 0.6. The only exceptions were 3 Friesians in which the tail circumference was slightly higher (26 cm in 1 horse and 27 cm in 2 horses), which could have led to slightly overestimated blood pressure. However, even when those 3 horses were excluded, MAP, SAP, DAP, and PP remained significantly higher in the Friesian horses. Only 1 of the 3 horses had considerably higher blood pressure (SAP, 182 mmHg; DAP, 106 mmHg; MAP, 134 mmHg), but also a high PP (76 mmHg). The latter is not influenced by width‐to‐tail circumference ratio and therefore suggests that the pressure differences were not a measurement artifact. Also, CO and systemic vascular resistance (SVR) may affect BP. Cardiac output however was not different between breeds. Systemic vascular resistance was not measured in our study, but increased SVR can lead to a higher DAP,^40^ which was found in these Friesian horses. On the other hand, stiffer arteries will result in higher SAP and PP,^31^, ^40^ which also was found in our study. Friesians might therefore have a combination of increased AWS and increased SVR.

Another reason why AWS parameters should be interpreted with caution is the different body conformation of Friesian horses compared to Warmblood horses. Although differences were relatively small, investigated Friesians had significantly smaller height at the withers compared to Warmbloods (162 ± 4 cm versus 167 ± 5 cm, respectively), whereas total body length (188 ± 11 cm versus 180 ± 9 cm, respectively) and chest circumference (199 ± 7 cm versus 195 ± 8 cm, respectively) were significantly larger. The smaller height at the withers of the Friesians might explain smaller blood vessel size. Because of this difference in diameter, relative stiffness parameters, such as arterial strain (S_A_, S_D_), distensibility coefficient (DC_A_, DC_D_) and stiffness index (SI), are preferred over absolute stiffness parameters, such as arterial diameter and area change (ΔD; ΔA) and compliance coefficient (CC_D_, CC_A_). The smaller arteries of Friesian horses, may lead to smaller absolute differences in area and lumen change during the cardiac cycle, which would wrongly suggest that arteries in Friesians are stiffer compared to Warmbloods when only absolute stiffness parameters would be considered. For the same reason, the PWV also should be interpreted with caution, because for the same CO, PWV will increase when arterial cross‐sectional area decreases.^41^

Our study had a number of limitations. A major limitation was that only a small number of Warmblood horses were used to study the relation between in vivo horse length and ex vivo determined distance between the investigated arteries, and that no Friesian horses were included in this part of the study. As mentioned previously, Friesian horses have a different body conformation compared to Warmblood horses. Friesians are not only smaller with a larger chest circumference, but they also are longer compared to Warmblood horses. Friesians may have different arterial lengths in relation to body length, which may affect the calculated PWV and thus regional AWS results. An ex vivo study with more horses and including a group of Friesian horses would allow better definition of breed specific data and also would account for anatomical variations.

Another limitation of our study is the use of noninvasively determined arterial blood pressure. A previous study showed that noninvasively collected SAP, MAP, and DAP correlated well with invasive measurements at the level of the facial or transverse facial artery, corrected to the level of the heart base, in the standing, normotensive, nonsedated horse.^31^ Results found in our study, both for Friesians and Warmblood horses, were within normal limits and thus can be considered representative of actual central blood pressure. In our study, coccygeal PP was used for calculation of aortic, common carotid, and external iliac artery local AWS parameters, but PP is known to vary over the arterial tree^29^ and might have been slightly different for the carotid artery, external iliac artery, and aorta compared to the coccygeal artery. Nevertheless, the same method was used in both breeds, making comparison possible.

Overall, we conclude that Friesians have higher arterial blood pressure compared to Warmblood horses, in combination with a stiffer aorta. The higher SAP and PP, in combination with a higher DAP suggests that Friesians might have a combination of increased AWS and increased SVR. Whether the increased blood pressure is the cause or a consequence of the increased aortic AWS cannot be concluded from our study. These findings, in combination with the previously shown differences in amount and cross‐linking pattern of collagen, make it highly probable that the predisposition of Friesians to aortic rupture is, at least partially, related to increased AWS of the aorta. Additional in vitro biomechanical testing, should provide more insight.

## CONFLICT OF INTEREST DECLARATION

Authors declare no conflict of interest.

## OFF‐LABEL ANTIMICROBIAL DECLARATION

Authors declare no off‐label use of antimicrobials.

## INSTITUTIONAL ANIMAL CARE AND USE COMMITTEE (IACUC) OR OTHER APPROVAL DECLARATION

The study was approved by the ethical committee of the Faculty of Veterinary Medicine and Bioscience Engineering (EC2018/111).

## HUMAN ETHICS APPROVAL DECLARATION

Authors declare human ethics approval was not needed for this study.
